# What Are the Remedies?

**Published:** 1889-06

**Authors:** 


### WHAT ARE THE REMEDIES?

What remedies have the following symptoms?

1. Sensation as if he would creep into his own body; he crouches together as much as he can, with pain in the thighs.

2. *After the chill*, thirst; drinking causes headache; tickling in larynx causes dry, continuous cough, which lasts through the heat; oppression of breathing, heaviness in middle of chest, with anxiety, abstaining from drinking, ameliorates all these symptoms; sweat relieves.

3. Sensation as if being lifted up high into air, tormented by anxiety, that slightest touch or motion would make her fall down from this height; headache.

4. Tetanic spasms from swallowing tobacco.

5. Pain as if all the bones were being torn to pieces, with vomiting and pain in the bowels.

6. Sciatica, sudden shooting, causing lameness; feels as if (left) hip-joint were wrenched; pain and lameness extend to popliteal space; worse from moderate, better from violent motion.

7. Pains shift about rapidly in phalanges and metacarpal bones.

8. Dares not remain fasting; better from warm diet.

9. Pinching, grasping pain in left hamstrings; worse at night, with night-sweat, and frequent urination.

10. Diarrhœa from drinking coffee; sugar aggravates pain in stomach, and wine causes headache.

